# Influence of the composition of high-strength concrete and mortar on the compressive fatigue behaviour

**DOI:** 10.1617/s11527-021-01868-7

**Published:** 2022-03-08

**Authors:** Nadja Oneschkow, Tim Timmermann

**Affiliations:** Institute of Building Materials Science, Leibniz University Hannover, Hannover, Germany

**Keywords:** High-strength concrete, Fatigue damage, Strain, Stiffness, Dissipated energy, Acoustic emission

## Abstract

The results of compressive fatigue investigations on four high-strength concretes and their corresponding mortars are presented. The influences of coarse aggregates generally, the substitution of basalt coarse aggregate by granite, the addition of silica fume and the variation of the water to cement (*w/c*) ratio are investigated systematically. The numbers of cycles to failure, the developments of strain, stiffness, dissipated energy and acoustic emission hits are focused on in the analyses. The results clearly show that coarse aggregates can influence the fatigue behaviour of concretes in a negative way at higher stress levels and in a positive way at lower stress levels compared to mortars. The granite coarse aggregate decreases the adverse effect at higher stress levels due to its lower modulus of elasticity compared to that of the basalt aggregate. Silica fume improves the fatigue behaviour of concrete and mortar strongly. The increase of the *w/c* ratio and, thus, the increase in porosity reduces the fatigue resistance of concrete and mortar significantly, due to the weakening of the mortar matrix and of the interfacial transition zone. The results demonstrate the interaction of the coarse aggregates and the mortar matrix with their specific properties, which leads to a certain fatigue behaviour. The acoustic emission gives additional valuable strain-independent information of the damage processes occurring, possibly also on micro- and nanoscales.

## Introduction

Concrete compositions with ever-higher compressive strengths enable the realisation of more filigree concrete structures. These structures are exposed to fatigue-relevant loads to a higher extent because of their lower ratio of deadweight to non-static loads. Moreover, special structures, such as wind energy turbines or machine foundations, are generally exposed to fatigue loading with huge numbers of load cycles. The development of concrete types with ever-higher compressive strengths and the expanded construction of wind energy turbines have led to increased research activities in the field of fatigue resistance of concrete in the last few decades. After focusing mainly on the numbers of cycles to failure, the focus has been more on the damage process in concrete in the last years (e.g. [1–3]), using damage indicators such as strain, stiffness or dissipated energy.

It is well-known that high-strength concrete has a denser binder matrix with increased compressive strength and improved interfacial transition zones (ITZ) compared to normal-strength concrete. This also leads to an increased modulus of elasticity. However, the influence of the improved microstructure on the compressive fatigue resistance has not yet been clarified. Contradictory results are documented in literature. Some investigators found that high-strength concrete has a lower fatigue resistance compared to normal-strength concrete (e.g. [4]). No or negligible influence was found in other investigations (e.g. [5–9]). However, consensus exists concerning differences in the well-known s-shaped strain development with three phases. High-strength concrete shows a longer second phase of the strain development compared to normal-strength concrete [1, 4, 9–11].

Only a few investigations were focused on the influence of the concrete composition and the porosity of the mortar matrix on the compressive fatigue behaviour. Mehmel and Kern [12] investigated the compressive fatigue behaviour of normal-strength concrete and of cement stone. They found that the interaction between the “solid phase” (coarse aggregates) and the “viscose phase” (cement stone) is essential for the fatigue behaviour of concrete. Furthermore, the change of the increasing branch of the hysteresis loop from a convex to a concave shape is characteristic for concrete and could not be detected for the cement stone. This change of shape of the hysteresis loop for concretes can be traced back to stiffening in the range of higher stresses and loosening in the range of lower stresses, which was confirmed by [2]. Shah and Chandra observed that the interaction between aggregates and cement stone is essential for the fatigue failure mode of concrete [13]. Furthermore, they found that specimens of cement stone did not show volume dilatation at the end of the fatigue process and, thus, differ strongly from concrete. They postulated two stages of crack growth, which depend on the maximum stress level applied. Stage I crack growth, without volume dilatation, occurs in concrete specimens, which did not fail, and in specimens of cement stone. Stage II crack growth, with accompanied volume dilatation, occurs in concrete before failure occurs. Mun et al. stated that the fatigue behaviour of concrete is influenced by the type of binder and by the compressive strength [14].

Regarding the influence of the type of coarse aggregate, Breitenbücher et al. [15] and Ibuk [16] found for a normal-strength concrete that the sensitivity towards compressive fatigue damage increases with increasing differences in the modulus of elasticity between coarse aggregate and hardened cement paste. However, no differences in the numbers of cycles to failure and the strain gradient in phase II could be found in [5] for a high-strength concrete with limestone or gravel as coarse aggregates.

Mehmel and Kern [12] and Lusche [17] understood the stiffer coarse aggregates as a disruption of the cement matrix in their model suggestion. They lead to an inhomogeneous stress distribution with areas of internal peak stresses, which are higher than the external subjected stresses. These areas are located in the cement matrix next to the coarse aggregates. The ratio of stiffness between the coarse aggregates and cement matrix as well as the size and volume of the aggregates influence the magnitude of the internal peak stresses. Mehmel and Kern assumed that these areas of internal peak stresses, caused by the heterogeneity of concrete, lead to damage and deterioration within the cement matrix in addition to the damage in the ITZ [12]. The results of Thiele [2] confirm a strong inhomogeneous three-dimensional stress distribution with a diffuse, spread compressive damaging effect in the cement matrix, and a localised, vertically orientated tensile damage. It is assumed that the compressive fatigue behaviour is strongly influenced by these compressive damage effects [2]. Results documented in [18] confirm the findings of [2, 12].

Vicente et al. [19] reported for a high-strength concrete containing air-entraining agent that higher porosity leads to a worse compressive fatigue resistance at the same stress level applied. Furthermore, they found that especially the bigger pores have a negative effect because of the creation of stress concentrations in the mortar matrix. Of course, these findings can only be hints because of the different characteristics of synthetically induced air pores compared to natural existing pores in the concrete microstructure. Zhang et al. found no influence of different water to cement (*w/c*) ratios on the bending fatigue resistance for a normal-strength concrete [20]. Weng et al. investigated normal-strength mortars with different *w/c* ratios regarding the acoustic emission activity due to monotonically increasing compressive loads [21]. The number of the acoustic emission hits (AE-hits) linearly decreases with an increasing *w/c* ratio. Weng et al. attributed this effect to the increased porosity, which may facilitate the growth of cracks and results in an earlier failure of the specimens.

It is well-known that silica fume reduces the porosity and the number and size of cracks in the ITZ and mortar matrix of the pristine concrete due to the filler effect, crystallising effects and the pozzolanic effect. Thus, it improves the strength of the ITZ and mortar matrix (e.g. [22–24]). Concerning the influence of silica fume on the compressive fatigue behaviour, no investigations could be found in literature. However, improved bending fatigue resistance was found in [22] for a high-strength concrete and in [25] for a high-strength self-compacting concrete with silica fume.

Indications are available that fatigue damage might be traced back to the formation and development of cracks in the mortar matrix and in the ITZ [13–15, 26]. Due to the diffuse compressive fatigue damage, which occurs on a very small scale, and due to the limited resolution in combination with limited sizes of investigable regions of interests, cracks are generally hardly reliably detectable with imaging techniques and mostly only in phase III of the strain development (e.g. [2, 13]). Even computer tomography often only enables the detection of relatively wide cracks shortly before failure due to the high density of the concrete and the necessary specimen diameters for fatigue testing (see e.g. [25, 27, 28]). However, Schaan et al. [29] found needle- or lath-shaped regions of lower density in the binder matrix of an ultra-high-strength concrete using transmission electron microscopy (TEM) and focused ion beam (FIB). This damage on the nanoscale increased in number within the compressive fatigue process.

Overall, the measuring and analysing of (macroscopic) damage indicators is of great importance for the fatigue research of concrete, due to the existing limitations of the imaging techniques. In addition to strain, stiffness and dissipated energy development, the acoustic emission is a continuously measurable damage indicator, which allows for additional and strain-independent information about the damage process in concrete’s microstructure [18, 30–33]. Overall, very little knowledge exists today about the influence of the concrete composition or, rather, the properties of concrete microstructure on the compressive fatigue behaviour.

In this paper, the results of compressive fatigue tests on four different, systematically modified concrete compositions and their corresponding mortars are presented. Two types of high-strength concrete with basalt, respectively, granite coarse aggregate, one high-strength concrete with added silica fume and one high-strength concrete with a higher *w/c* ratio, respectively, higher porosity of microstructure are analysed. The fatigue behaviour of their corresponding hardened mortar matrix, called ‘mortar’, is analysed comparatively, investigating the influence of coarse aggregates. The numbers of cycles to failure, the developments of strain, stiffness, dissipated energy and AE-hits are focused on in the analyses. The results presented were determined within a conjoint research project together with Prof. Löhnert, TU Dresden, which is part of the DFG Priority Programme SPP 2020.

## Experimental programme

### Materials and specimens

#### Concrete composition and mortars

Four high-strength concretes were investigated (Table 1). Concrete RH1-B is the reference composition in this project and in the Priority Programme SPP 2020 with a *w/c* ratio of 0.35, basalt aggregate and without silica fume. Concrete RH1-B-Si has the same composition as RH1-B but the cement was partially replaced by silica fume, following the approach of the same paste film thickness of [34]. In addition, a high-strength concrete RH1-B-w with an increased *w/c* ratio of 0.5 and a lower compressive strength was considered. This composition is slightly further adjusted in order to ensure similar fresh concrete properties. Based on the reference composition, the basalt aggregate was substituted by granite aggregate with the same mass content and the same grain size distribution in concrete RH1-G. The densities of the aggregates were experimentally determined as 2.9 kg/dm^3^ for the basalt and as 2.7 kg/dm^3^ for the granite. Thus, the volume of the granite aggregate in RH1-G is with 105% slightly larger than the volume of the basalt aggregate in RH1-B.

**Table 1:** Concrete compositions

Component	RH1-B	RH1-B-Si	RH1-B-w	RH1-G
Portland Cement (CEM I 52.5 R HS/NA) [kg/m^3^]	500	447	461	500
Microsilica [kg/m^3^]	–	45	–	–
Quartz sand (0/0.5 mm) [kg/m^3^]	75	75	69	75
Sand (0/2 mm) [kg/m^3^]	850	850	783	850
Basalt (2/5 mm) [kg/m^3^]	350	350	322	–
Basalt (5/8 mm) [kg/m^3^]	570	570	525	–
Granite (2/5 mm) [kg/m^3^]	–	–	–	350
Granite (5/8 mm) [kg/m^3^]	–	–	–	570
Superplasticiser [kg/m^3^]	5.00	4.90	0.49	5.00
Stabiliser [kg/m^3^]	2.85	2.80	–	2.85
*w/c* ratio; *w/c*_eq_ ratio [–]	0.35	0.35	0.50	0.35

In addition, corresponding mortars were investigated (Table 2). The mortars were obtained by sieving off the concrete components with grain sizes  ≥ 2 mm. Concrete RH1-G was included to investigate the influence of different types of aggregates on the fatigue behaviour of concrete. Thus, no corresponding mortar of RH1-G was included in the investigation. Because mortar M-B could contain a small volume of basalt particles smaller than 2 mm, comparison is not drawn between M-B and RH1-G.

**Table 2:** 28-day compressive strength, modulus of elasticity and compressive fatigue reference strength

Concrete	RH1-B	RH1-B-Si		RH1-B-w	RH1-G
	B1	B1	B2	B1	B1
*f*_cm,cube_ [MPa]	113	133	129	71	109
*E*_cm_ [MPa]	40,000	44,400	–^*^	33,700	35,600
*f*_cm,ref_ [MPa]	96	123	120	58	110

Mortar	M-B	M-B-Si		M-B-w	
	B1	B1		B1	
*f*_cm,cube_ [MPa]	108	114		78	
*E*_cm_ [MPa]	34,900	38,200		23,000	
*f*_cm,ref_ [MPa]	97	116		75	

*Not determined

The compressive strengths of the aggregates were tested using drill cores with a height of *h* = 180 mm and a diameter of *d* = 60 mm. The test velocity was 0.5 MPa/s. The modulus of elasticity was determined according to DIN EN 12390-13 [35] using drill cores with *h* = 300 mm and *d* = 150 mm. The basalt has a mean compressive strength of *f*_cm,b_ = 326 MPa and a mean modulus of elasticity of *E*_m,b_ = 99,800 MPa. The values are *f*_cm,g_ = 203 MPa and *E*_m,g_ = 56,200 MPa for the granite. Thus, a high difference in the compressive strength and, especially, in the modulus of elasticity exists, as it was intended by the selection of the aggregate types investigated. The mean values of the 28-day compressive strength *f*_cm,cube_ determined according to DIN EN 12390-3 [36] and the modulus of elasticity *E*_cm_ according to DIN EN 12390-13 [35] of the four concretes and three mortars are summarised in Table 2.

With regard to the mortars, silica fume leads to a slight increase of the 28-day compressive strength *f*_cm,cube_ and an increase of the modulus of elasticity *E*_cm_ (M-B-Si). Due to the higher *w/c* ratio of 0.5, the mortar M-B-w has a significantly lower 28-day compressive strength and a significantly lower modulus of elasticity compared to the reference mortar M-B. These changes in properties can be traced back to the higher porosity of M-B-w.

Regarding the concretes, the basalt aggregate in RH1-B increases the compressive strength *f*_cm,cube_ only slightly compared to the mortar M-B. However, it increases the modulus of elasticity compared to that of the mortar. The silica fume leads to an increase of the compressive strength and modulus of elasticity of RH1-B-Si compared to RH1-B. Whereby the silica fume has an enhanced positive effect on the compressive strength of the concrete compared to that of the mortar, revealing its strengthening effect in the ITZ of the coarse aggregates and in highly stressed areas of the mortar matrix. The compressive strength *f*_cm,cube_ of RH1-B-w is 63% and the modulus of elasticity *E*_cm_ 84% of that of the reference concrete RH1-B due to the higher *w/c* ratio. The basalt coarse aggregate in RH1-B-w leads to an increased modulus of elasticity but the compressive strength slightly decreases compared to the mortar. The substitution of basalt aggregate by granite aggregate has a negligible effect on the compressive strength but the modulus of elasticity is 89% of that of RH1-B due to the lower modulus of elasticity of the granite aggregate. The properties displayed in Table 2 demonstrate that the target manipulation of properties by the modified compositions was successful for the case of monotonically increasing loading.

The compressive fatigue reference strengths of the concretes and mortars *f*_cm,ref_ were determined just before conducting the fatigue tests, using five specimens from the same batch and with the same geometry as the specimens used in the fatigue tests. These tests were conducted force-controlled with a test velocity of 0.5 MPa/s. The compressive fatigue reference strength *f*_cm,ref_ was calculated for each concrete and mortar as the mean value of its compressive strengths. The compressive fatigue reference strength is required to determine the axial test stresses based on the chosen fatigues stress levels.

#### Specimens

The investigations were conducted using cylindrical specimens with a height of *h* = 180 mm and diameter of *d* = 60 mm. PVC formwork was used for casting. The concrete was filled into the formworks in two equal layers and each layer was mechanically compacted using a vibrating table. The formwork of the concrete specimens was removed 7 days after concreting and the cylinders were stored in standard climate (20 °C/65% R.H.) until testing. The treatment of the mortar specimens was different compared to that of the concrete specimens in order to avoid increased microcracking due to shrinkage: the mortar specimens were also stored in standard climate until testing but the formwork was removed 14 days after concreting. Subsequently, these specimens were stored wrapped in plastic foil for 14 days and then without foil until testing for at least further 28 days in standard climate (gentle drying). The test surfaces of all specimens were plane-parallel ground and polished to achieve a uniform stress distribution. Due to the corona virus shutdown of the laboratory, the specimens of batch 1 (B1) of the concrete with silica fume had to be tested later than originally planned with an age of specimens between 126 and 147 days. Therefore, batch 2 (B2) was produced and additional fatigue tests were conducted. The age of all specimens at testing, except of those from batch 1, was between 56 and 87 days.

### Test programme and experimental setup

The maximum stress level in the fatigue tests was either *S*_max_ = 0.85 or *S*_max_ = 0.70. The minimum stress level was kept constant at *S*_min_ = 0.05 in all tests. The test frequency applied was *f*_t_ = 1.0 Hz in all fatigue tests. This test frequency was chosen in order to prevent specimens heating due to mechanical loading. The full amplitude was applied in the first load cycle. Three specimens of each concrete and mortar type were loaded at each stress level until fatigue failure occurred. Exception was made for the concrete with silica fume of batch 2 at *S*_max_ = 0.70 where two run-outs occurred. Overall, 47 specimens were tested in the fatigue investigations presented in this paper. An overview of the fatigue tests is available from Table 3 in the appendix.

**Fig. 1:**
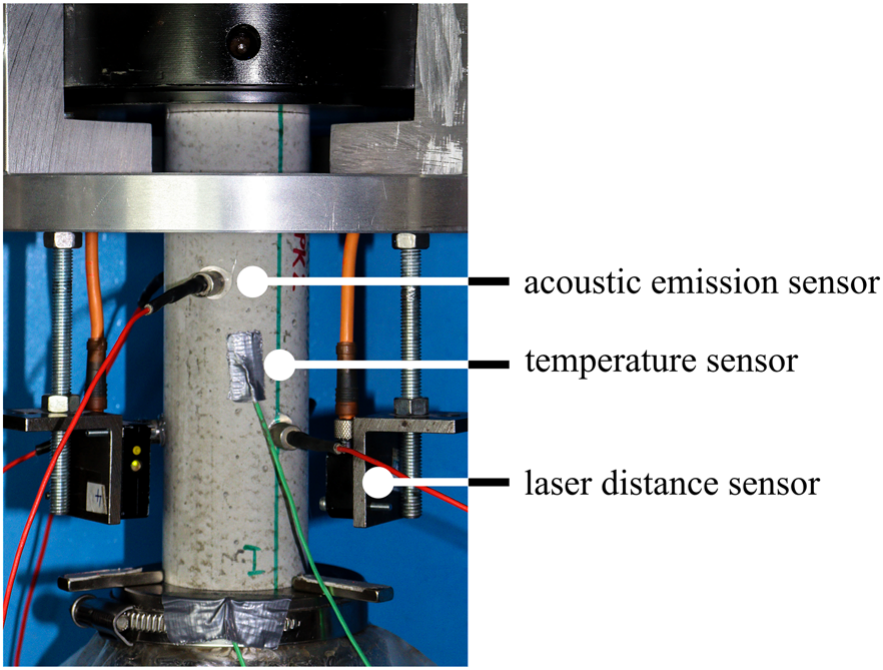
Experimental setup [18]

The fatigue tests were carried out force-controlled using a class 0.5 servo-hydraulic testing machine with a 500 kN actuator (according to ISO 7500-1 [37]). The axial deformations were measured continuously using three laser distance sensors positioned on the circumference of the specimen at angles of 0°, 120° and 240°, see Fig. 1. In addition, the axial force, the axial displacement of the actuator and the temperature of specimen’s surface at mid-height, the ambient temperature and the temperature of the loading plate were measured. The sampling rate was 300 Hz for all measured quantities. For the acoustic emission analysis, six sensors were attached to the specimens. The sensors with a wideband frequency response within the range of 250–1600 kHz were positioned at 60° from one another, alternating in the upper and lower third of the specimen. Based on pre-tests, a threshold of 40 dB was defined to separate the useful signal from background noise.

### Analysing techniques

As explained previously, two batches of the concrete with silica fume RH1-B-Si had to be investigated. At first, the developments of the damage indicators were comparatively evaluated for the specimens of both batches. This evaluation demonstrated good agreement at the higher stress level *S*_max_ = 0.85. However, flatter developments were determined for the specimens of batch 2 at *S*_max_ = 0.70 leading to higher numbers of cycles and test break-offs (run-out specimens). Additionally considering the similar values of compressive strengths of both batches (cf. Table 2) it was decided to analyse the test results of both batches together at *S*_max_ = 0.85. At *S*_max_ = 0.70, a quantitative analysis of damage indicators is not reliably possible due to the break-offs. Thus, the two run-out specimens of batch 2 could not be included in the analyses.

In the following, the fatigue damage indicators maximum and minimum strain, stiffness, dissipated energy and AE-hits are analysed. These analyses include all cycles up to and including the last full cycle before failure. This approach was established due to the occurrence of fatigue failure at different stresses (but mostly in the range of the peak stress) and due to the high-grade instable state of the microstructure, which is reached in the last cycle (cf. also [11]). For this paper, the gradients of strain, stiffness, dissipated energy and cumulated AE-hits in phase II were determined between fixed values of *N/N*_f_ = 0.20 and *N/N*_f_ = 0.80 for the reason of better comparability (note: different approach compared to previous papers [33, 38]).

The peak strains were obtained by peak analyses of the sinusoidal strain curves determined by the three laser distance sensors and subsequently averaged in order to obtaining the mean strain development for each specimen. The gradient of the strain development in phase II (i.e. strain increase per load cycle) and the total growth of strain within the degradation process are focused on as parameters. The development of stiffness was determined for each specimen based on the secant modulus in the decreasing branch of the hysteresis loop, according to Eq. (1), using the mean strains of each specimen.

1 $${{E}}_{{\text{S}}}= \frac{{ \sigma }_{{\max}}-{ \sigma }_{{\min}}}{{\varepsilon}_{{\max}}-{\varepsilon}_{{\min}}}$$

2 $$\Delta{{E}}_{{\text{S}}}^{0.0-1.0}= \frac{{{E}}_{{\text{S}}}^{0.0}-{{E}}_{{\text{S}}}^{1.0}}{{{E}}_{{\text{S}}}^{0.0}}$$

Regarding the development of stiffness, the gradient of stiffness development in phase II (i.e. reduction of stiffness per load cycle) and the percentile reduction of stiffness from the first to the last load cycle (cf. Eq. (2)), with the initial value of stiffness in the first cycle as reference, are analysed in the following.

The dissipated energy was calculated for each load cycle as the area enclosed by the hysteresis loop. The development of dissipated energy describes the visco-elastic material behaviour within the fatigue process and, thus, the deformation capability. Here, the gradient of dissipated energy in phase II (i.e. dissipated energy per load cycle), the average dissipated energy per load cycle in phase II and the sum of dissipated energy within the complete fatigue process are analysed as parameters. The average dissipated energy per load cycle in phase II gives information about the range of deformation capability within the fatigue process for the purpose of comparison between different concretes and mortars. It is calculated as follows:

3 $${\text{av} }\,{{E}}_{{\text{D}}}^{0.2-0.8}{=}\frac{\sum {{E}}_{{\text{D}}}^{0.2-0.8}}{{{N}}^{0.2-0.8}}$$

The AE-hits are summarised from cycle to cycle and displayed as developments of cumulated AE-hits. The gradient of cumulated AE-hits in phase II (i.e. increase in AE-hits per load cycle) and the total number of AE-hits in the complete fatigue process are used as parameters.

In view of the multitude of single curves, averaged curves of the damage indicators were determined for each concrete and mortar for the purpose of graphical presentation. They were calculated for identical tests by averaging the developments of each damage indicator with regard to the related numbers of load cycles *N/N*_f_ and, subsequently, multiplying this averaged curve by the mean value of the number of cycles to failure *N*_f_. Please note that the gradients of the curves are distorted due to this approach. Thus, the developments in Figs. 3, 4, 5, 6, 7, 8, 9 and 10 display the relative (higher/lower) but not the absolute relation between the different concretes and mortars. For the quantitative analyses, the mean values of the parameters given in Tables 4 and 5 in the appendix are used, which are calculated based on the single developments.

## Experimental results and discussion

### Numbers of cycles to failure

**Fig. 2:**
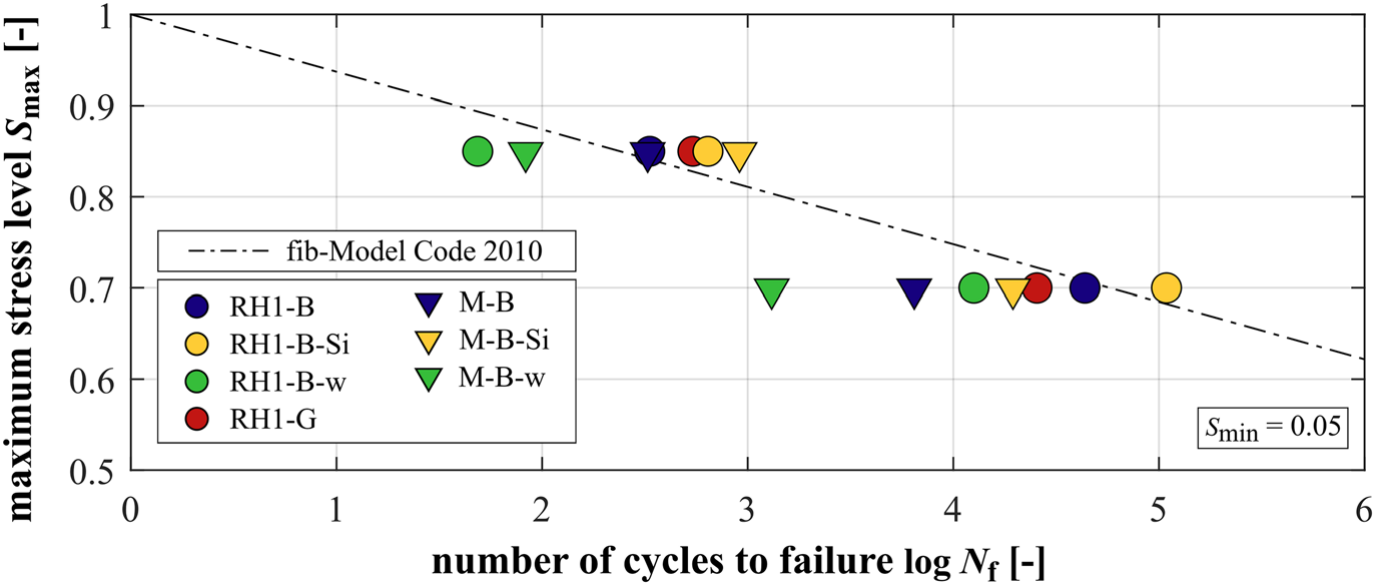
Mean numbers of cycles to failure of the concretes and mortars

The mean numbers of cycles to failure of the concretes and mortars are presented in Fig. 2 as logarithmic values (log *N*_f_). The numbers of cycles applied of the two run-out specimens of RH1-B-Si are not included in the appropriate mean value. All single and mean values are included in Table 3 in the appendix.

The mortar with silica fume M-B-Si shows the highest mean numbers of cycles to failure followed by the mortar M-B and the mortar M-B-w. Thus, the addition of silica fume has a positive effect on the mortars’ fatigue resistance and the higher *w/c* ratio has a negative effect. However, the increase in mean number of cycles to failure of the mortar with silica fume is accompanied by a higher scatter of single values (cf. Table 3). The concretes show the same order in mean numbers of cycles to failure as their mortars. For the concrete with silica fume RH1-B-Si, the increase is also accompanied by an increase in the scatter of single results (Table 3). The substitution of basalt aggregate by granite leads to contradictory results. A higher mean number of cycles to failure at the higher stress level *S*_max_ = 0.85 and a lower one at the lower stress level *S*_max_ = 0.70 can be seen for RH1-G compared to RH1-B.

However, the numbers of cycles to failure of the concretes at *S*_max_ = 0.85 are nearly equal (RH1-B) or lower (RH1-B-Si and RH1-B-w) than those of the corresponding mortars. By contrast, at *S*_max_ = 0.70, all concretes reached significantly higher mean numbers of cycles to failure compared to their corresponding mortars. Regarding the concrete with silica fume, the difference in mean numbers of cycles to failure is higher than displayed in Fig. 2 due to the run-outs that are not included. It can also be seen that the increase of numbers of cycles to failure due to the reduction of the stress level is more pronounced for the concretes.

### Development of strain

**Fig. 3:**
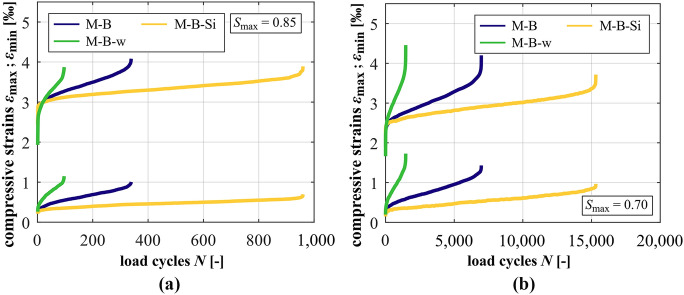
Averaged developments of strain of the mortars at *S*_max_ = 0.85 (**a**) and *S*_max_ = 0.70 (**b**)

**Fig. 4:**
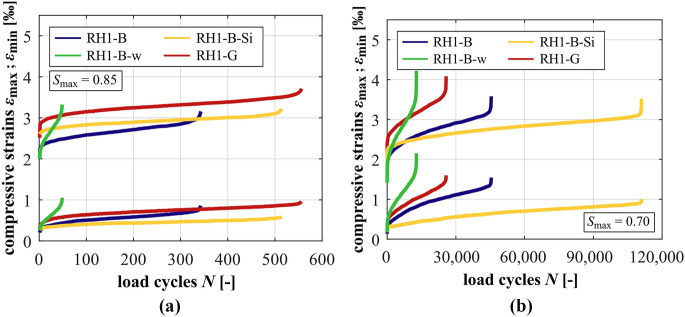
Averaged developments of strain of the concretes at *S*_max_ = 0.85 (**a**) and *S*_max_ = 0.70 (**b**)

The developments of strain of the mortars at *S*_max_ = 0.85 and *S*_max_ = 0.70 are shown in Fig. 3a and b. In Fig. 4, those of the concretes at *S*_max_ = 0.85 (a) and *S*_max_ = 0.70 (b) are displayed. It is obvious that the mortar with the higher *w/c* ratio M-B-w shows the steepest gradients of strain in phase II, followed by the reference mortar M-B and the mortar with silica fume M-B-Si with the flattest gradient in phase II at both stress levels (cf. also Table 5). The total growth of strain of the mortar M-B-w is the highest, followed by M-B and M-B-Si, although the numbers of cycles to failure of M-B-w are the smallest, followed by M-B and M-B-Si. The similar values of the total growth of maximum strain at *S*_max_ = 0.85 of M-B-w and M-B is an exception (cf. Table 5), whereby the number of cycles to failure of M-B-w is considerably lower (cf. Figure 2). With decreasing stress level, the gradients in phase II decrease and the total growth of strain increases for each mortar due to the higher numbers of cycles to failure. The results clearly show that the mortar is strengthened due to the silica fume and weakened due to the higher *w/c* ratio.

From the strain developments of the concretes, it is obvious that the granite aggregate and silica fume lead to higher initial maximum strains compared to the reference concrete RH1-B, especially at *S*_max_ = 0.85. This can be traced back to the ratio of the change in compressive strength and the change in modulus of elasticity due to the adjustment of the concrete composition, see Table 2. Similar to the mortars, the concrete RH1-B-w has the steepest gradient of maximum and minimum strain in phase II, followed by the reference concrete RH1-B and the concrete with silica RH1-B-Si at both stress levels (cf. Table 4). Comparing concretes RH1-B and RH1-G, it is obvious that the concrete with granite aggregate has a significantly flatter gradient in phase II at *S*_max_ = 0.85 but this relation is reversed at *S*_max_ = 0.70. Thus, the granite aggregate leads to an improved concrete behaviour at the higher stress level. This is reversed at the lower stress level. Despite the differing numbers of cycles to failure and the reversed relation of the strain gradients, the total growth of strain of RH1-B and RH1-G is quite similar at both stress levels. This is an indication that the total growth of strain is influenced to a higher extent by the mortar’s fatigue behaviour rather than by the interaction of the coarse aggregates with the surrounding mortar matrix, respectively, the ratio of stiffness.

Comparing the strain developments of the concretes with their corresponding mortars, the gradients in phase II of the concretes are flatter at *S*_max_ = 0.70, meaning a smaller strain increase per load cycle. Regarding *S*_max_ = 0.85, the concrete with silica fume and the concrete with the higher *w/c* ratio show steeper gradients compared to their corresponding mortars, whereby the difference between the concrete and mortar with silica fume is very small. Thus, the presence of basalt coarse aggregate has adverse effects at the higher stress level and is beneficial at the lower stress level in both cases. By contrast, the concrete RH1-B shows a considerably flatter gradient compared to its corresponding mortar M-B at the higher stress level although RH1-B and M-B reached similar mean numbers of cycles to failure (cf. Figure 2). This comparatively steeper gradient of the mortar specimens might be traced back to the larger volume of mortar matrix compared to the concrete specimens, which is damaged due to fatigue loading. This aspect will be further discussed in Section 4.1.

However, in the case of the mortar and concrete with silica fume, the mortar specimens are strengthened to a higher degree. The gradient of strain is more decreased (M-B → M-B-Si) in comparison to the corresponding concrete (RH1-B → RH1-B-Si). Thus, the relation of gradients in phase II of the mortar and the concrete is reversed compared to that of RH1-B and M-B (without silica fume). In the case of the higher *w/c* ratio, the inhomogeneous stress distribution caused by the basalt coarse aggregates affects the surrounding weakened mortar to a higher extent. Thus, the gradient in phase II of the concrete is increased more strongly (RH1-B → RH1-B-w) compared to that of the mortar (M-B → M-B-w). Therefore, the relation of gradients in phase II of the mortar and the concrete is also reversed compared to the relation of RH1-B and M-B. All mortars have higher magnitudes of maximum strain compared to their corresponding concretes, whereby the differences depend on the type of mortar. However, these findings reveal the interaction of the coarse aggregates and the mortar with their specific properties, which leads to a certain strain development.

### Development of stiffness

**Fig. 5:**
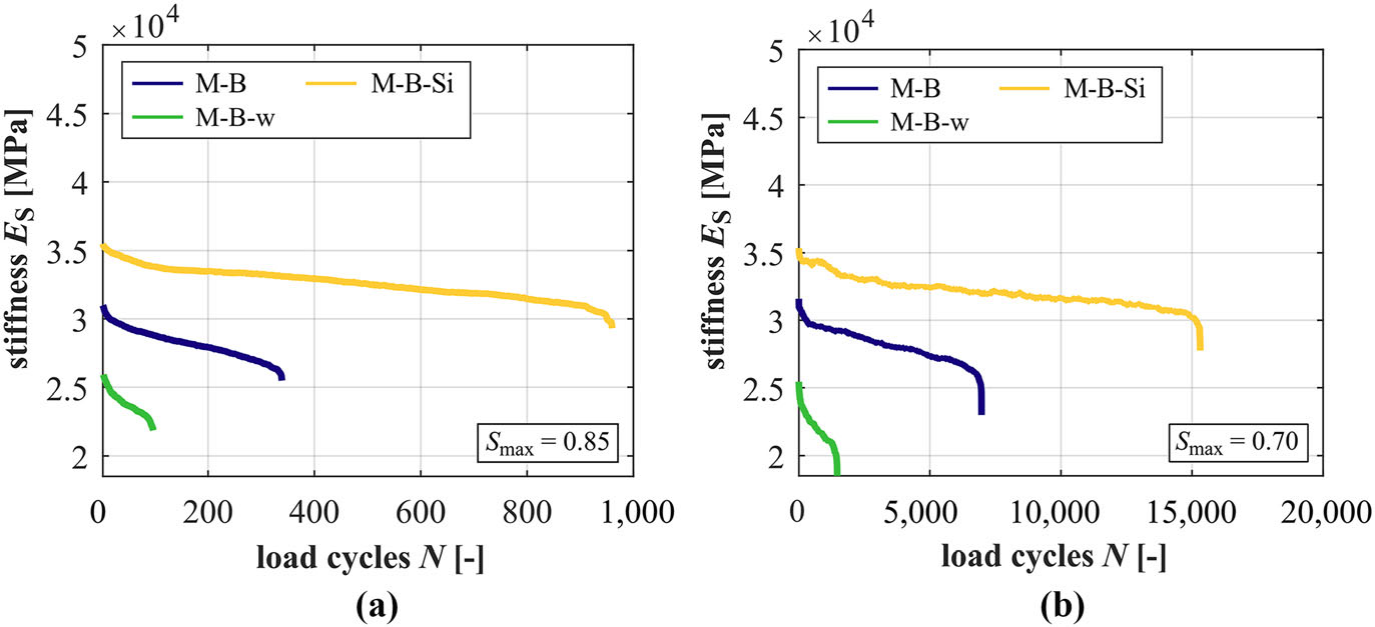
Averaged developments of stiffness of the mortars at *S*_max_ = 0.85 (**a**) and *S*_max_ = 0.70 (**b**)

**Fig. 6:**
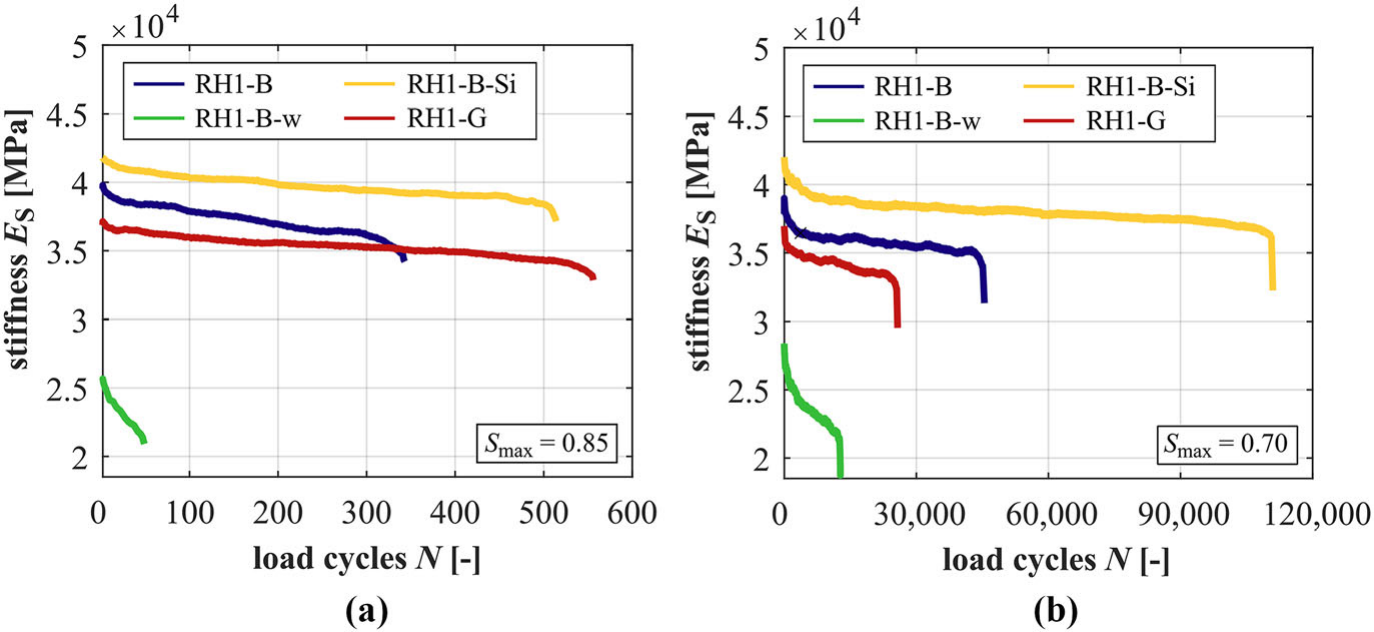
Averaged developments of stiffness of the concretes at *S*_max_ = 0.85 (**a**) and *S*_max_ = 0.70 (**b**)

The developments of stiffness of the mortars are shown in Fig. 5 for both stress levels *S*_max_ = 0.85 and *S*_max_ = 0.70 and those for the concretes in Fig. 6. The decrease of stress level leads to flatter gradients of stiffness of the concretes and mortars. The differences are the smallest between the mortar, respectively, concrete with silica fume and the highest between the mortar, respectively, concrete with the higher *w/c* ratio. It is obvious from Fig. 5 that M-B-w has the lowest magnitude of stiffness, followed by M-B and the mortar with silica fume M-B-Si, which is consistent with the relation of the modulus of elasticity (cf. Table 2). The gradient in phase II of M-B-w is the steepest, followed by M-B and M-B-Si at both stress levels, meaning the highest stiffness degradation per cycle for M-B-w (cf. Table 5). These differences between the mortars are higher at the higher stress level.

Regarding the concretes, the concrete RH1-B-w with the higher *w/c* ratio shows the steepest gradients of stiffness in phase II, followed by the reference concrete RH1-B and the concrete with silica fume RH1-B-Si at both stress levels, thus, the same as for the mortars. However, at *S*_max_ = 0.70, the difference between the reference concrete and the concrete with silica fume and, thus, the effect of silica fume is small (cf. Table 4). It must be mentioned that the gradients of stiffness of the two run-out specimens (which could not be considered) are flatter compared to the value given in Table 4 and, therefore, would decrease the (mean) value of the gradient of stiffness displayed. At *S*_max_ = 0.85, a further indication exists that the granite aggregate has a positive effect on the degradation mechanism, leading to a flatter gradient in phase II of RH1-G compared to the concrete RH1-B. However, the gradient in phase II of RH1-G is slightly steeper than that of RH1-B at *S*_max_ = 0.70, revealing that the positive effect does not exists on this stress level. Here, the reversal is less pronounced compared to the gradient of strain (Section 3.2). The percentile reduction of stiffness is the highest for the concrete RH1-B-w, followed by the concrete RH1-B and the concrete with silica fume RH1-B-Si at the higher stress level. At the lower stress level, concrete RH1-B-w and concrete RH1-B-Si show higher percentile reductions of stiffness compared to the reference concrete. Thus, the percentile reduction of stiffness depends on the concrete composition and a general limit criterion for different concretes is questionable.

Comparing the stiffness developments of the concretes with those of the corresponding mortars, all concretes show steeper gradients of stiffness in phase II at *S*_max_ = 0.85 and flatter gradients in phase II at *S*_max_ = 0.70. Thus, the basalt coarse aggregate seems to increase the reduction of stiffness per load cycle at the higher stress level and decrease it at the lower stress level. The difference in gradients of stiffness is the lowest between RH1-B-Si and M-B-Si and the highest between RH1-B-w and M-B-w. Thus, the effect of the basalt coarse aggregate on the stiffness reduction per load cycle is the smallest for the concrete with strengthened mortar and ITZ (silica fume) and the highest for the concrete with the weakened mortar and ITZ (higher *w/c* ratio).

### Development of dissipated energy

**Fig. 7:**
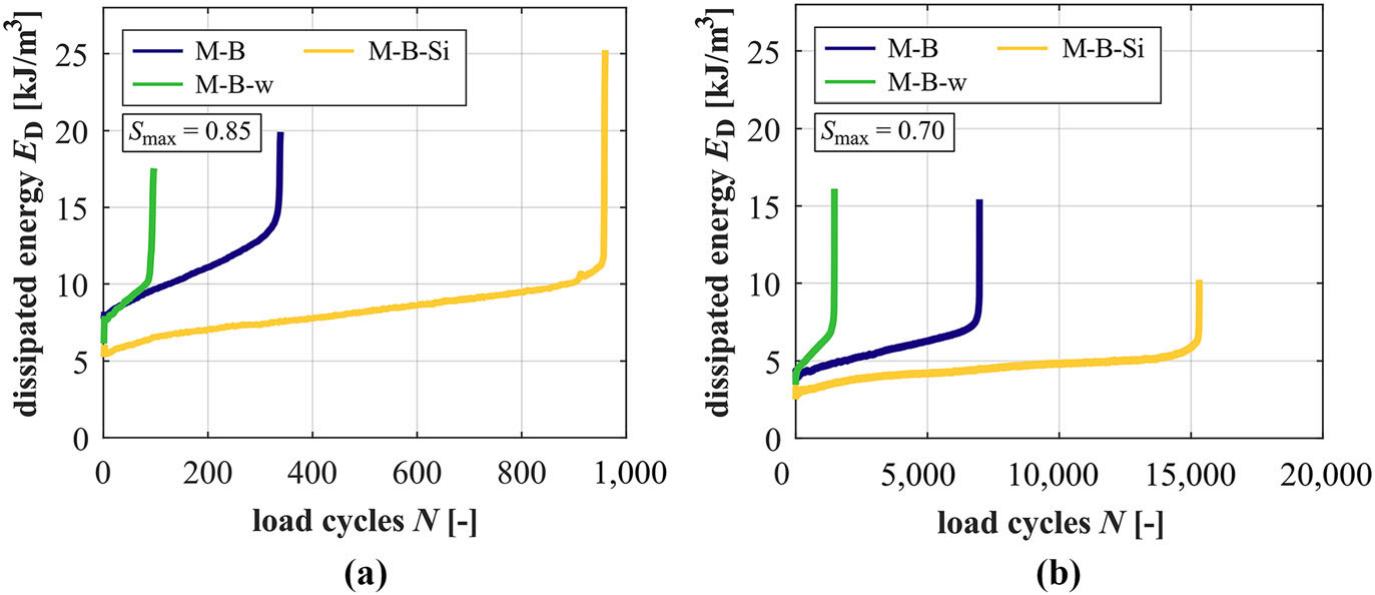
Averaged developments of dissipated energy of the mortars at *S*_max_ = 0.85 (**a**) and *S*_max_ = 0.70 (**b**)

**Fig. 8:**
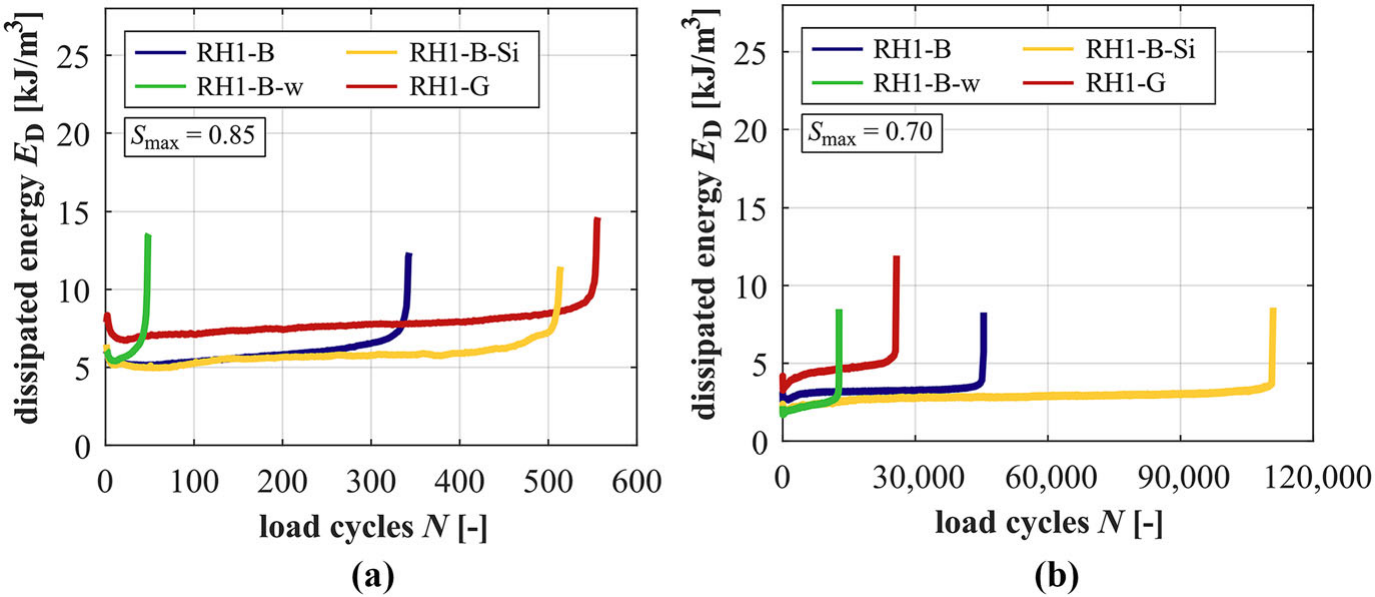
Averaged developments of dissipated energy of the concretes at *S*_max_ = 0.85 (**a**) and *S*_max_ = 0.70 (**b**)

The developments of the dissipated energy within the fatigue process are shown for the mortars in Fig. 7 and for the concretes in Fig. 8 for both stress levels. The gradients in phase II of dissipated energy are generally steeper at the higher stress level, but the sum of dissipated energy is much lower due to the significantly lower numbers of cycles to failure (cf. Tables 4 and 5). M-B-w has the steepest gradients and the lowest sums of dissipated energy and M-B-Si has the flattest gradient and highest sum of dissipated energy at both stress levels.

Considering the concretes, the gradient in phase II of RH1-B-w is the steepest, followed by RH1-B and RH1-B-Si at *S*_max_ = 0.85 and, thus, the same order as for the mortars exists. Regarding *S*_max_ = 0.70, the highest value of the gradient is also reached by RH1-B-w, followed by RH1-B and RH1-B-Si with similar values (the latter would be further reduced by the run-out specimens). Compared to the basalt concrete RH1-B, the granite concrete RH1-G shows a flatter gradient in phase II at *S*_max_ = 0.85, but the relation is reversed at *S*_max_ = 0.70, same as detected for the gradient of strain (cf. Section 3.2). Similar to the mortars, RH1-B-w dissipates the lowest sum of energy, followed by the reference concrete and the concrete with silica fume at both stress levels.

It can be seen for RH1-B-Si and *S*_max_ = 0.85 in Fig. 8a that the development is somewhat wavy in phase II (more pronounced for the single developments as visible in Fig. 8a). This represents shifts between decrease and increase of the areas of the hysteresis loops within the fatigue process. This kind of progression was found for all RH1-B-Si specimens tested at this stress level and not for those of the corresponding mortar. Thus, it differs from the developments of all other concretes and mortars. Therefore, it is suggested to be characteristic for the concrete with silica fume at this stress level. This wavy development must be traced back to the (positive) effect of silica fume in the mortar matrix and in the ITZ of the concrete at the higher stress level. Furthermore, it must also be related to the inhomogeneous stress distribution caused by the basalt coarse aggregate.

The gradients of dissipated energy in phase II are steeper for the mortars compared to their corresponding concretes. Exception exists regarding M-B-w and RH1-B-w at *S*_max_ = 0.85. Here, the gradient for the mortar is flatter. The decrease of the stress level reduces the gradients of dissipated energy of the concretes to a higher extent compared to those of the mortars. Concerning the lower stress level, the difference in gradients is the highest between M-B-w and RH1-B-w by far. Regarding the average dissipated energy in phase II, it is interesting that the average dissipated energy of the mortars is always higher compared to that of their corresponding concretes at both stress levels, meaning a more pronounced deformation capability without coarse aggregate.

### Development of acoustic emission hits

**Fig. 9:**
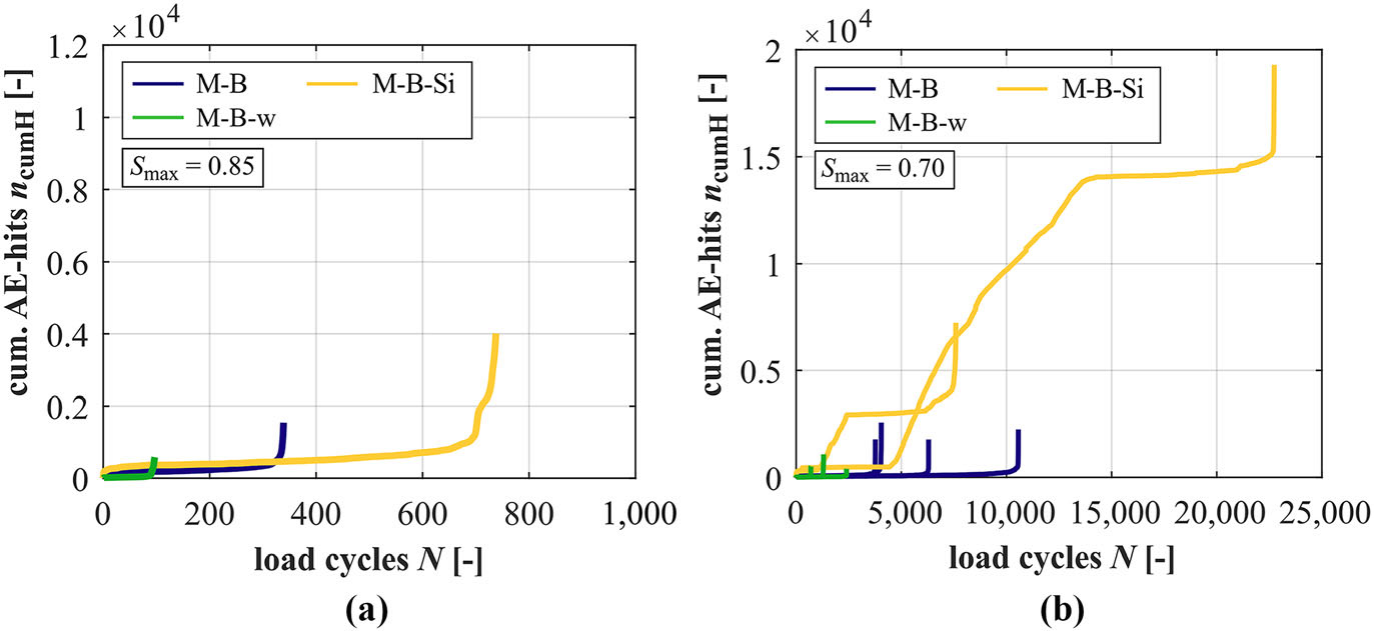
Averaged developments of cumulated AE-hits of the mortars at *S*_max_ = 0.85 (**a**) and developments of cumulated AE-hits of the mortars at *S*_max_ = 0.70 (**b**)

**Fig. 10:**
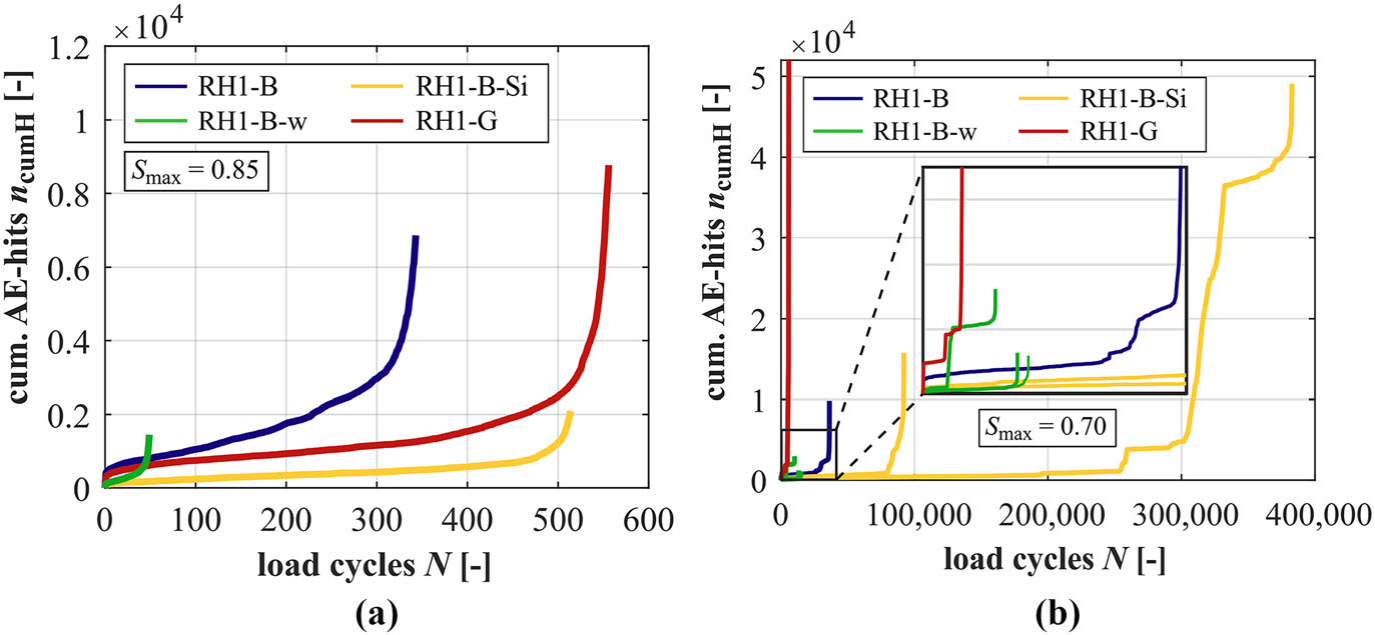
Averaged developments of cumulated AE-hits of the concretes for *S*_max_ = 0.85 (**a**) and developments of cumulated AE-hits of the concretes at *S*_max_ = 0.70 (**b**)

Unfortunately, single AE-sensors detached from the test specimen surface in single tests. Therefore, measurements of AE-hits are not available for all of the tests conducted. The developments of cumulated AE-hits are shown for the mortars in Fig. 9 and in Fig. 10 for the concretes. For *S*_max_ = 0.70, the averaged curves of cumulated AE-hits cannot be meaningfully determined because the differences in single curves are too large. Therefore, single curves are displayed in Figs. 9b and 10b. In Fig. 10b, the developments are zoomed out in a box for small load cycles *N*.

It is obvious that the mortar M-B-Si has the steepest gradient of cumulated AE-hits in phase II together with the largest total numbers of hits, followed by M-B and M-B-w with the flattest gradients and the lowest total numbers of hits at *S*_max_ = 0.85 (cf. also Table 5). Thus, the relation of gradients of the mortars is not plausible in a first view, considering the positive effect of the silica fume, respectively, the negative effect of the higher *w/c* ratio, which were determined based on the other damage indicators and on the numbers of cycles to failure (Fig. 2). Furthermore, an extreme total number of AE-hits was detected at the lower stress level for RH1-G (see Table 4). These AE-hits show different characteristics compared to those of the other concretes, as described in [32]. The cyclic loading at this stress level seems to induce a kind of ‘crackling’ [32].

Both findings reveal that the comparative analyses of parameters based solely on numbers of AE-hits is not sufficient for the comparison of different mortars, or rather, different concretes with different mortar matrixes. The results obtained emphasise that the characteristics of the AE-signals have to be considered additionally in future analyses. Therefore, the analysis is limited to the effect of the stress levels and the general development of cumulated AE-hits in the following.

It is obvious from Tables 4 and 5 that the total numbers of AE-hits are generally smaller at the higher stress level compared to the lower stress level for the concretes and mortars. The gradients of cumulated AE-hits are steeper at the higher stress level, whereby they are only determinable for the mortars M-B and M-B-w at both stress levels. Figure 10a shows that the increase of AE-hits in the last phase is considerably less pronounced for the concrete RH1-B-Si compared to the developments of the other concretes. This differs from the characteristics of the mortars in Fig. 9a.

At the stress level *S*_max_ = 0.70, stepwise developments of cumulated AE-hits after a first initial phase are observable in almost all tests carried out on the concretes and on the mortar with silica fume (Figs. 9b and 10b). Furthermore, this stepwise development is most pronounced for the concrete with silica fume. The rather horizontal sections represent relatively low acoustic activity in relation to the strong increase before and afterwards. Concurrently, these steps are not visible in the other (macroscopic) damage indicators investigated (cf. Sections 3.2–3.4). Thus, this might indicate damage processes occurring on very small scales (maybe sub-microscale) in the sections with a strong increase in AE-hits, which are not yet visible macroscopically. In the rather horizontal sections (temporary) equilibrium states of these damage processes on a very small scale might be reached within the microstructure.

## Discussion

In this section the results previously described are discussed superiorly, also considering results and model assumptions documented in literature. By bringing them together, an idea of the processes in the microstructure forms, which helps to understand the damage mechanisms. As noticeable in the previous sections, the properties of the coarse aggregate and the mortar matrix interact. This interaction together with the relative volume of coarse aggregate and mortar matrix in the concrete specimens lead to the superimposition of effects, which finally leads to a certain macroscopically detectable fatigue behaviour of the concrete.

### Influence of basalt coarse aggregate

The influence of the basalt coarse aggregate can be found by comparing the concretes with basalt coarse aggregate (RH1-B, RH1-B-Si and RH1-B-w) with their corresponding mortars (M-B, M-B-Si, M-B-w). It should be noted that own fatigue tests with comparable loads conducted on the basalt stone showed no indications of damage occurring.

At the lower stress level, the basalt coarse aggregate in concrete leads to an improved fatigue behaviour observable by the flatter gradients of strain, stiffness and dissipated energy in phase II and by the higher numbers of cycles to failure of all concretes. At the higher stress level, the gradients of stiffness are steeper for all concretes and, thus, more stiffness reduction per load cycle occurs in the concretes, indicating a negative effect. Regarding the gradient of strain in phase II, the concrete with silica fume RH1-B-Si and that with the higher *w/c* ratio RH1-B-w also have higher values compared to their corresponding mortars. They also reached lower numbers of cycles to failure compared to their corresponding mortars. These findings are also an indication of a negative effect of the basalt coarse aggregate at higher stress levels due to the induced inhomogeneous stress distribution, described previously in [2, 12].

By contrast, the reference concrete RH1-B has a flatter gradient of strain compared to the corresponding mortar M-B and, furthermore, reached almost the same mean number of cycles to failure at *S*_max_ = 0.85. Considering the overall view of the results obtained for the other concretes and mortars and considering that the gradients of stiffness show the same relation for RH1-B/M-B as for the other concretes/mortars, the following explanation is assumed: the concrete specimens of RH1-B, of course, contain a smaller volume of mortar matrix compared to the mortar specimens M-B. If a comparable stress distribution was present, then quantitatively less fatigue-induced damage would develop in the mortar matrix of the concrete. But concurrently a more inhomogeneous stress distribution compared to the (pure) mortar specimens is present at *S*_max_ = 0.85 due to the basalt coarse aggregate. This might lead to the development of more damage, especially, in highly stressed areas of the mortar matrix. Both effects superimpose and lead to the macroscopically detectable fatigue behaviour of the concrete.

Both effects are generally present for all concretes but the damage sensitivity of the mortars differs, due to the addition of silica fume (RH1-B-Si/M-B-Si), respectively, the increased *w/c* ratio (RH1-B-w/M-B-w). As a result, the relation of the gradients of strain and of the numbers of cycle to failure are different for these concretes/mortars compared to the reference concrete/mortar. Altogether, it is assumed that the similarity of the numbers of cycles to failure of RH1-B and M-B at *S*_max_ = 0.85 is a rather random result.

The comparison of the average dissipated energy and the gradient of dissipated energy in phase II between the concretes and their mortars demonstrates, that the deformation capability and its increase due to the induced damage is reduced by the basalt coarse aggregate. The first aspect is connected to the higher stiffness of the coarse aggregate compared to the mortar matrix. The latter aspect must be connected to the smaller volume of the mortar matrix in the concrete, which is damaged. Exception was found for the gradient of dissipated energy of RH1-B-w and M-B-w at the higher stress level, which is steeper for the concrete. This might be related to a relatively strong damage development in the weakened mortar matrix due to the presence of coarse aggregate. Nevertheless, an increase of deformation capability due to the presence of basalt coarse aggregate seems to be conceivable in general, considering additional displacements between the coarse aggregates and the surrounding mortar matrix. Unfortunately, this cannot be differentiated based on the investigation conducted.

Overall, the developments of strain and dissipated energy of concrete seem to be strongly influenced by the volume of and the damage development in the mortar matrix. The gradient of stiffness in phase II might be more influenced by the interaction of the basalt coarse aggregates and the surrounding mortar matrix. The effect of coarse aggregate could be predominated by the generation of an inhomogeneous stress distribution with highly stressed areas, especially at higher stress levels, which accelerate the damage processes in the mortar matrix and in the ITZ. At lower stress levels, smaller numbers of highly stressed areas and/or lower stresses in such areas can be assumed. Here, a predominantly beneficial effect exists due to the higher fatigue resistance and stiffness of the coarse aggregate compared to the surrounding mortar matrix, both leading to an attraction of stresses. An effect of the differing quantities of (weaker) ITZ in the concretes in comparison to the mortars, cannot be distinguished due to the superimposition of effects previously described.

### Influence of the properties of coarse aggregate

The substitution of basalt aggregate by granite aggregate with a considerably lower modulus of elasticity benefits the concrete’s fatigue behaviour at higher stress levels and impairs the behaviour at lower stress levels. This results in flatter gradients of strain, stiffness and dissipated energy in phase II of concrete RH1-G compared to the reference concrete RH1-B at the higher stress level and in a reversed relation at the lower stress level. The volume of both coarse aggregates used is slightly different (cf. Section 2.1.1). Of course, the strength of the ITZ or, rather, the strength of bond between the mortar matrix and the aggregates could also be slightly different. However, in the view of the authors, this cannot explain the phenomena observed. Therefore, the beneficial effect at the higher stress level compared to the effect of basalt aggregate is traced back to the reduction of inhomogeneous stress distribution. This is caused by the smaller difference between the modulus of elasticity of the surrounding mortar matrix and that of the granite aggregate (cf. Section 2.1.1), whose effect is especially pronounced at higher stress levels. Thus, the results basically confirm the suggestions from [2, 12]. At the lower stress level, this positive effect (reduction of inhomogeneous stress distribution) of the granite aggregate is less pronounced. Here, the higher fatigue resistance and/or the higher modulus of elasticity of the basalt aggregate seems to be beneficial for the fatigue resistance of the concrete.

For the higher stress level *S*_max_ = 0.85, the obtained results indicate a higher fatigue sensitivity of concrete with aggregates with higher modulus of elasticity as stated in [15, 16]. In contrast, this higher sensitivity is shown in [15, 16] at a lower maximum stress level of *S*_max_ = 0.675 based on the stiffness, which was determined under (slower) monotonically increasing loading. At the similar stress level of *S*_max_ = 0.70, a reversed influence is found here. However, in contrast to the investigation presented here, normal-strength concretes were under investigation in [15, 16].

The ‘crackling’ of the granite concrete RH1-G at the lower stress level, found by analyses of the characteristics of the AE-signals and previously reported in [32], might indicate, on the one hand, that the granite aggregates are more highly stressed at the lower stress level compared to the higher stress level, although the maximum stress subjected externally is lower. On the other hand, this ‘crackling’ could also arise from the damaged matrix around the coarse aggregates. Both aspects could be caused by processes of internal stress redistribution that differ at both stress levels and, furthermore, are dependent on the ratio of stiffness of the coarse aggregate and the mortar matrix and, of course, on the damage sensitivity of the surrounding mortar matrix. Further analyses of the characteristics of the AE-signals are necessary. An approach was presented in [18] and is currently under further development. The granite aggregate with its lower stiffness seems to increase the deformation capability of the concrete additionally, which was observed by the average dissipated energy.

### Influence of silica fume

The results obtained clearly demonstrate that the addition of the silica fume leads to a better fatigue resistance of the concrete and mortar at high and low stress levels. This can be traced back to its strengthening effect in the mortar matrix and in the ITZ. Based on the comparison of the mortars (M-B → M-B-Si), respectively, concretes (RH1-B → RH1-B-Si), it becomes clear that the gradients of strain and stiffness in phase II are flatter due to the silica fume. The basalt coarse aggregate in the concrete leads to slightly steeper gradients of strain and stiffness at the higher stress level (M-B-Si → RH1-B-Si). Concurrently, the strengthening effect on the gradient of stiffness of the concrete and mortar is more pronounced at the higher stress level (stress level dependency). Thus, the effect of the silica fume seems to be related to the magnitude of stresses occurring in the microstructure and, therefore, also to the stress distribution in the concrete caused by the coarse aggregates.

Based on the average dissipated energy and on the gradient of dissipated energy in phase II, it is observable that the silica fume additionally reduces the deformation capability and the increase in deformation capability due to fatigue damage of the concrete and mortar. These effects are more pronounced for the mortar specimens. The development of the dissipated energy of the concrete with silica fume at the higher stress level, representing shifts between the increase and decrease of the area of the hysteresis loop, is an indication of the occurrence of macroscopically measurable temporal equilibrium states. The fact that this kind of development was not detected for the corresponding mortar indicates a connection to the presence of coarse aggregates.

The stepwise development of cumulated AE-hits at the lower stress level might represent damage processes on a very small scale (maybe sub-microscale), which are not macroscopically measurable. It is more pronounced for the concrete and mortar with silica fume compared to the other concretes and mortars investigated. Thus, the strengthening of the mortar, and perhaps also of the ITZ, due to the silica fume seems to improve the capability to reach equilibrium states of that damage processes on a very small scale. Overall, the results reveal that a strong positive influence of the silica fume exists on the concrete’s and mortar’s fatigue behaviour. It has to be assumed that the silica fume also improves the fatigue resistance of the ITZ, although this effect could not be distinguished within the investigations conducted.

### Influence of porosity

The increase of the *w/c* ratio and, thus, the higher porosity in the mortar matrix and the concurrently weaker ITZ clearly lead to a worse fatigue behaviour of the concrete and mortar. Here, the difference in modulus of elasticity between the mortar and the basalt aggregate is the highest (cf. Table 2). Compared to the reference concrete and mortar, the gradients of strain, stiffness and dissipated energy of the mortar and concrete are considerably steeper (M-B → M-B-w; RH1-B → RH1-B-w). The difference depends on the stress level. Thus, a more pronounced strain increase, stiffness reduction and increase in deformation capability per load cycle occurred due to the higher porosity of the microstructure.

Furthermore, regarding the higher stress level, the presence of basalt coarse aggregate has an additional negative effect on the fatigue behaviour of the concrete compared to that of the mortar (M-B-w → RH1-B-w). This results in steeper gradients of strain, stiffness and dissipated energy. In contrast, at the lower stress level, the gradients of the concrete are flatter. Both effects (positive and negative) of the coarse aggregate previously described in Section 4.1 are significantly more pronounced compared to the other concretes, visible by the higher differences in values between RH1-B-w and M-B-w. Thus, the predominantly negative effect of basalt coarse aggregate at higher stress levels due to the generation of a more inhomogeneous stress distribution is more pronounced due to the weakened mortar matrix and ITZ. Concurrently, at lower stress levels, the positive effect is also more pronounced due to the significantly higher fatigue resistance and higher stiffness compared to the weakened mortar matrix and ITZ and, thus, a stronger attraction of stresses. Consequently, the coarse aggregate leads to an accelerated development of damage in the mortar matrix and the ITZ at higher stress levels and to a more pronounced stress relief and less damage in the mortar matrix and ITZ at lower stress levels. The fatigue resistance of the high-strength concrete and mortar is significantly reduced by the higher porosity of the microstructure.

## Summary and conclusion

The results of compressive fatigue investigations on four different compositions of high-strength concrete and their corresponding mortars were presented and discussed comparatively in this paper. For this purpose, the concrete compositions were systematically adjusted in order to analyse the influence of coarse aggregate in general, of two types of coarse aggregate (basalt and granite), the addition of silica fume and the increase of the *w/c* ratio, i.e. the porosity of the microstructure. Two maximum stress levels of *S*_max_ = 0.85 and *S*_max_ = 0.70 with the same minimum stress level of *S*_min_ = 0.05 and a test frequency of *f*_t_ = 1.0 Hz were investigated. The developments of strain, stiffness, dissipated energy and cumulated AE-hits were analysed comparatively as damage indicators. Each influence was investigated separately in order to pinpoint each effect on the fatigue behaviour. The results of the influence of the composition of high-strength concrete and mortar can be summarised as follows:

- Basalt coarse aggregate impairs the fatigue behaviour of high-strength concrete at higher stress levels compared to that of the mortar due to the inhomogeneous stress distribution with more highly stressed areas in the microstructure, which accelerate the damage processes in the mortar matrix and in the ITZ. Basalt coarse aggregate improves the fatigue behaviour of high-strength concrete at lower stress levels compared to that of the mortar, due to its higher fatigue resistance and/or higher stiffness compared to the mortar matrix. Here, the attraction of stresses, which relieves stresses in the mortar matrix, might be of importance. However, the effect of coarse aggregate on the concrete’s fatigue behaviour depends on the difference in fatigue resistance and stiffness between the coarse aggregate and the surrounding mortar matrix.
- Granite aggregate, with its lower modulus of elasticity compared to basalt aggregate, might reduce the inhomogeneous stress distribution, especially at higher stress levels, and, thus, influences the fatigue behaviour of high-strength concrete in a positive way. This effect is reduced with decreasing stress levels. Furthermore, granite aggregate might increase the deformation capability of high-strength concrete compared to basalt aggregate.
- The silica fume can improve the fatigue behaviour of high-strength concrete and mortar to a high extent. The influence of added silica fume depends on the original properties of the mortar matrix and ITZ and on the internal stress distribution due to the interaction of the coarse aggregate and the mortar matrix.
- A higher *w/c* ratio or, rather, higher porosity of the microstructure impairs the fatigue behaviour of high-strength concrete and mortar to a high extent. This must be traced back to the weakening of the mortar matrix and the ITZ.
- The worse fatigue behaviour of the mortars at lower stress levels can be traced back to the smaller relative volume of basalt aggregate with its higher fatigue resistance and higher stiffness.
- Regarding high-strength concretes, the damage indicator gradient of strain in phase II is predominated by the damage processes in the mortar matrix and the volume of the mortar matrix. The gradients of stiffness and dissipated energy in phase II are more influenced by the interaction of coarse aggregate and mortar matrix.
- The number of AE-hits is a good parameter for the comparative evaluation of the damage activity of concretes and their corresponding mortar. Regarding the comparison of concretes with different mortar matrix, the characteristics of the AE-signals should be considered in addition to their number.

The investigations presented lead to new knowledge about the fatigue behaviour of concretes and mortars with respect to their compositions. By the analysis of acoustic emissions, additional strain-independent knowledge can be generated, whereby the characteristics of AE-signals should be considered more detailed in future analyses. The clustering of AE-hits [18] seems to be a promising way that should be further developed. Overall, the results clearly show that the damage propagation depends to a high extent on the properties of the coarse aggregate and mortar matrix as well as on the stress distribution in the microstructure, which itself is influenced by the damage propagation. For a better understanding of the damage processes in the microstructure, numerical models are necessary, which enable insights into the microstructure-dependent damage development.

## Appendix

See Tables 3, 4 and

**Table 3:** Single and mean numbers of cycles to failure

Concrete	RH1-B	RH1-B-Si		RH1-B-w	RH1-G
	B1	B1	B2	B1	B1
0.85/0.05	431	412	815	41	449
	351	148	674	58	730
	246	562	474	49	488
Mean	343	514		49	556
0.70/0.05	64,209	90,625	275,804 (r)	14,825	28,839
	35,871	92,368	138,966 (r)	10,181	27,458
	36,267	149,828	–	13,277	20,960
Mean	45,449	110,941	–	12,761	25,752

Mortar	M-B	M-B-Si		M-B-w	
	B1	B1		B1	
0.85/0.05	427	727		44	
	224	1403		75	
	366	749		172	
Mean	339	960		97	
0.70/0.05	4048	7597		2398	
	6301	23,026		1308	
	10,566	42,384		703	
Mean	6972	24,336		1470	

(r) run-out specimen

**Table 4:** Concretes: mean values of parameters of damage indicators

Concrete		RH1-B	RH1-B-Si	RH1-B-w	RH1-G
0.85/0.05	$\Delta{\varepsilon}_{{\max}}^{0.0{-}1.0}$ [‰]	0.94	0.63	1.21	0.91
	$\Delta{\varepsilon}_{{\min}}^{0.0{-}1.0}$ [‰]	0.64	0.31	0.76	0.62
	${\text{grad} }\,{\varepsilon}_{{\max}}^{0.2{-}0.8}$ [–]	1.40 × 10^–6^	0.72 × 10^–6^	17.00 × 10^–6^	0.80 × 10^–6^
	${\text{grad} }\,{\varepsilon}_{{\min}}^{0.2{-}0.8}$ [–]	0.80 × 10^–6^	0.45 × 10^–6^	10.00 × 10^–6^	0.50 × 10^–6^
	$\Delta{{E}}_{{\text{S}}}^{0.0{-}1.0}$ [%]	14.60	11.77	19.52	12.15
	${\text{grad} }\,{{E}}_{{\text{S}}}^{0.2{-}0.8}$ [MPa]	− 10.82	− 4.44	− 73.27	− 3.31
	*ΣE*_D_ [kJ/m^3^]	2.02 × 10^3^	2.87 × 10^3^	0.30 × 10^3^	4.32 × 10^3^
	${\text{av} }\,{{E}}_{{\text{D}}}^{0.2{-}0.8}$ [kJ/m^3^]	5.70	5.70	5.85	7.61
	${\text{grad} }\,{{E}}_{{\text{D}}}^{0.2{-}0.8}$ [kJ/m^3^]	5.10 × 10^–3^	2.90 × 10^–3^	39.70 × 10^–3^	2.50 × 10^–3^
	*Σn*_H_ [–]	6854	2095	1450	8769
	${{{\text{grad}} \, n}}_{{\text{cumH}}}^{0.2{-}0.8}$ [–]	9.05	1.05	9.65	3.30
0.70/0.05	$\Delta{\varepsilon}_{{\max}}^{0.0{-}1.0}$ [‰]	1.77	1.39	2.71	1.79
	$\Delta{\varepsilon}_{{\min}}^{0.0{-}1.0}$ [‰]	1.39	0.78	1.96	1.32
	${\text{grad} }\,{\varepsilon}_{{\max}}^{0.2{-}0.8}$ [–]	1.90 × 10^–8^	0.67 × 10^–8^	10.00 × 10^–8^	3.60 × 10^–8^
	${\text{grad} }\,{\varepsilon}_{{\min}}^{0.2{-}0.8}$ [–]	1.70 × 10^–8^	0.53 × 10^–8^	8.00 × 10^–8^	3.10 × 10^–8^
	$\Delta{{E}}_{{\text{S}}}^{0.0{-}1.0}$ [%]	20.08	23.84	35.89	20.67
	${\text{grad} }\,{{E}}_{{\text{S}}}^{0.2{-}0.8}$ [MPa]	− 0.03	− 0.02	− 0.28	− 0.09
	*ΣE*_D_ [kJ/m^3^]	146.13 × 10^3^	313.96 × 10^3^	29.37 × 10^3^	118.73 × 10^3^
	${\text{av} }\,{{E}}_{{\text{D}}}^{0.2{-}0.8}$ [kJ/m^3^]	3.22	2.87	2.28	4.62
	${\text{grad} }\,{{E}}_{{\text{D}}}^{0.2{-}0.8}$ [kJ/m^3^]	0.63 × 10^–5^	0.73 × 10^–5^	5.70 × 10^–5^	3.80 × 10^–5^
	*Σn*_H_ [–]	10,086	32,683	1819	11,816,617
	${{{\text{grad}}\, n}}_{{\text{cumH}}}^{0.2{-}0.8}$ [–]	–*	–*	–*	–*

*Not determinable

**Table 5:** Mortars: mean values of parameters of damage indicators

Mortar		M-B	M-B-Si	M-B-w
0.85/0.05	$\Delta{\varepsilon}_{{\max}}^{0.0{-}1.0}$ [‰]	1.34	1.02	1.32
	$\Delta{\varepsilon}_{{\min}}^{0.0{-}1.0}$ [‰]	0.75	0.45	0.82
	${\text{grad} }\,{\varepsilon}_{{\max}}^{0.2{-}0.8}$ [–]	2.41 × 10^−6^	0.57 × 10^−6^	9.74 × 10^−6^
	${\text{grad} }\,{\varepsilon}_{{\min}}^{0.2{-}0.8}$ [–]	1.43 × 10^−6^	0.24 × 10^−6^	6.46 × 10^−6^
	$\Delta{{E}}_{{\text{S}}}^{0.0{-}1.0}$ [%]	19.03	17.75	16.83
	${\text{grad} }\,{{E}}_{{\text{S}}}^{0.2{-}0.8}$ [MPa]	− 9.03	− 3.57	− 29.50
	*ΣE*_D_ [kJ/m^3^]	3.66 × 10^3^	7.84 × 10^3^	0.90 × 10^3^
	${\text{av} }\,{{E}}_{{\text{D}}}^{0.2{-}0.8}$ [kJ/m^3^]	10.68	8.07	9.04
	${\text{grad} }\,{{E}}_{{\text{D}}}^{0.2{-}0.8}$ [kJ/m^3^]	15.60 × 10^−3^	4.30 × 10^−3^	35.40 × 10^−3^
	*Σn*_H_ [–]	1543	4365	586
	${{{\text{grad}}\, n}}_{{\text{cumH}}}^{0.2{-}0.8}$ [–]	0.71	1.58	0.40
0.70/0.05	$\Delta{\varepsilon}_{{\max}}^{0.0{-}1.0}$ [‰]	1.99	1.38	2.36
	$\Delta{\varepsilon}_{{\min}}^{0.0{-}1.0}$ [‰]	1.21	0.76	1.48
	${\text{grad} }\,{\varepsilon}_{{\max}}^{0.2{-}0.8}$ [–]	16.60 × 10^−8^	4.95 × 10^−8^	89.80 × 10^−8^
	${\text{grad} }\,{\varepsilon}_{{\min}}^{0.2{-}0.8}$ [–]	11.60 × 10^−8^	3.40 × 10^−8^	64.97 × 10^−8^
	$\Delta{{E}}_{{\text{S}}}^{0.0{-}1.0}$ [%]	27.69	22.00	31.21
	${\text{grad} }\,{{E}}_{{\text{S}}}^{0.2{-}0.8}$ [MPa]	− 0.60	− 0.23	− 2.50
	*ΣE*_D_ [kJ/m^3^]	38.83 × 10^3^	72.08 × 10^3^	8.72 × 10^3^
	${\text{av} }\,{{E}}_{{\text{D}}}^{0.2{-}0.8}$ [kJ/m^3^]	5.69	4.53	5.70
	${\text{grad} }\,{{E}}_{{\text{D}}}^{0.2{-}0.8}$ [kJ/m^3^]	48.9 × 10^−5^	16.0 × 10^−5^	214.4 × 10^−5^
	*Σn*_H_ [–]	2318	13,272	698
	${{{\text{grad}}\, n}}_{{\text{cumH}}}^{0.2{-}0.8}$ [–]	0.014	–*	0.043

*Not determinable

5.

## Meaning of symbols

*d* [mm]: Diameter of specimen
*ΣE*_D_ [kJ/m^3^]: Total sum of dissipated energy, *N/N*_f_ = 0.0–1.0
${\text{av} }\,{{E}}_{{\text{D}}}^{0.2{-}0.8}$ [kJ/m^3^]: Average dissipated energy in phase II between *N/N*_f_ = 0.2–0.8
${\text{grad} }\,{{E}}_{{\text{D}}}^{0.2{-}0.8}$ [kJ/m^3^]: Gradient of dissipated energy development in phase II between *N/N*_f_ = 0.2–0.8
*E*_cm_ [MPa]: Mean value of modulus of elasticity of concretes and mortars
*E*_S_ [MPa]: Stiffness in case of fatigue loading
$\Delta{{E}}_{{\text{S}}}^{0.0{-}1.0}$ [%]: Percentile reduction of stiffness between *N/N*_f_ = 0.0–1.0
${\text{grad} }\,{{E}}_{{\text{S}}}^{0.2{-}0.8}$ [MPa]: Gradient of stiffness development between *N/N*_f_ = 0.2–0.8
*f*_cm_ [MPa]: Mean value of 28-day compressive strength
*f*_cm,cube_ [MPa]: Mean value of 28-day compressive strength, 100 mm cube
*f*_cm,ref_ [MPa]: Fatigue reference compressive strength
*f*_t_ [Hz]: Test frequency
*h* [mm]: Height of specimen
*N* [–]: Number of cycles applied
*N*_f_ [–]: Number of cycles to failure
*N/N*_f_ [–]: Related number of cycles applied
*Σn*_H_ [–]: Total number of AE-hits, *N/N*_f_ = 0.0–1.0
${{{\text{grad}}\, n}}_{{\text{cumH}}}^{0.2-0.8}$ [–]: Gradient of cumulated AE-hits development in phase II between *N/N*_f_ = 0.2–0.8
*S*_max_ [–]: Maximum compressive stress level, *S*_max_ = *σ*_max_*/f*_cm,ref_
*S*_min_ [–]: Minimum compressive stress level, *S*_min_ = *σ*_min_*/f*_cm,ref_

## Meaning of Greek letters

*ε*_max_ [‰]: Strain at maximum peak stress
*ε*_min_ [‰]: Strain at minimum peak stress
$\Delta{\varepsilon}_{{\max}}^{0.0{-}1.0}$ [‰]: Total growth of maximum strain, *N/N*_f_ = 0.0–1.0
$\Delta{\varepsilon}_{{\min}}^{0.0{-}1.0}$ [‰]: Total growth of minimum strain, *N/N*_f_ = 0.0–1.0
${\text{grad} }\,{\varepsilon}_{{\max}}^{0.2-0.8}$ [–]: Gradient of max. strain development in phase II between *N/N*_f_ = 0.2–0.8
${\text{grad} }\,{\varepsilon}_{{\min}}^{0.2-0.8}$ [–]: Gradient of min. strain development in phase II between *N/N*_f_ = 0.2–0.8
*σ*_max_ [MPa]: Maximum peak stress (at maximum stress level)
*σ*_min_ [MPa]: Minimum peak stress (at minimum stress level

## References

[1] Oneschkow N (2014) Analyse des Ermüdungsverhaltens von Beton anhand der Dehnungsentwicklung. [Analysis of the fatigue behaviour of concrete with respect to the development of strain] (in German). Doctoral Thesis, Reports of the Institute of Building Materials Science, 13, Leibniz Universität Hannover. 10.15488/357

[2] Thiele M (2016) Experimentelle Untersuchungen und Analyse der Schädigungsevolution in Beton unter hochzyklischen Ermüdungsbeanspruchungen. [Experimental investigation and analysis of the damage development in concrete subjected to high-cycle fatigue] (in German). Doctoral Thesis, BAM-Dissertationsreihe Band 140, Technische Universität Berlin. 10.15488/357

[3] Song Z, Frühwirt T, Konietzky H (2018). Characteristics of dissipated energy of concrete subjected to cyclic loading. Constr Build Mater.

[4] Kim J-K, Kim Y-Y (1996). Experimental study of the fatigue behavior of high strength concrete. Cem Concr Res.

[5] Hordijk DA, Wolsink GM, de Vries J (1995). Fracture and fatigue behaviour of a high strength limestone concrete compared to gravel concrete. HERON.

[6] Kono S, Hasegawa H, Mori K, Ichioka Y, Sakashita M, Watanabe, F (2008) Low cycle fatigue characteristics of high strength concrete. In: Proceedings of the 8th international symposium on utilization of high-strength and high-performance concrete, Tokyo, pp 616–622

[7] Oneschkow N, Lohaus L (2017). Zum Nachweis von druckschwellbeanspruchtem Beton, Teil 2: Sicherheitsüberlegungen und Potenzial für Weiterentwicklungen. [About the fatigue design concept of concrete subjected to pure compressive fatigue loading. Part 2: Safety considerations and potential for further developments] (in German). Beton- und Stahlbetonbau.

[8] Basaldella M, Oneschkow N, Lohaus L (2021). Influence of the specimen production and preparation on the compressive strength and the fatigue resistance of HPC and UHPC. Mater Struct.

[9] Petković G, Stemland H, Rosseland S (1992) High strength concrete SP 3—Fatigue, Report 3.2: fatigue of high strength concrete. SINTEF Structural Engineering – FCB, Trondheim

[10] Hohberg R (2004) Zum Ermüdungsverhalten von Beton [About the fatigue behaviour of concrete] (in German). Doctoral Thesis, Technische Universität Berlin

[11] Oneschkow N (2016). Fatigue behaviour of high-strength concrete with respect to strain and stiffness. Int J Fatigue.

[12] Mehmel A, Kern E (1962) Elastische und plastische Stauchungen von Beton infolge Druckschwell- und Standbelastung [Elastic and plastic strains of concrete due to compressive fatigue and constant loading] (in German). Deutscher Ausschuss für Stahlbeton, Heft 153, Wilhelm Ernst & Sohn, Berlin

[13] Shah SP, Chandra S (1970). Fracture of concrete subjected to cyclic and sustained loading. ACI J.

[14] Mun J-S, Yang K-H, Kim S-J (2016). Tests on the compressive fatigue performance of various concretes. J Mater Civ Eng.

[15] Breitenbücher R, Ibuk H, Yüceoglu S (2008). Beeinflusst die Kornsteifigkeit der Gesteinskörnung im Beton den Degradationsprozess infolge zyklischer Druckbeanspruchung? [Does the aggregate stiffness in concrete affect the degradation process due to cyclic compressive loading?] (in German). Beton- und Stahlbetonbau.

[16] Ibuk H (2008) Ermüdungsverhalten von Beton unter Druckschwellbeanspruchung [Fatigue behaviour of concrete subjected to compressive fatigue loading] (in German). Doctoral Thesis, Ruhr Universität Bochum

[17] Lusche M (1971) Beitrag zum Bruchmechanismus von auf Druck beanspruchtem Normal- und Leichtbeton mit geschossenem Gefüge [Contribution to the fracture mechanism of compression loaded normal and lightweight concrete with closed structure] (in German). Doctoral Thesis. Ruhr-Universität Bochum

[18] Oneschkow N, Scheiden T, Hüpgen M, Rozanski C, Haist M (2021). Fatigue-induced damage in high-strength concrete microstructure. Materials.

[19] Vicente MA, González DC, Mínguez J, Tarifa MA, Ruiz G, Hindi R (2018). Influence of the pore morphology of high strength concrete on its fatigue life. Int J Fatigue.

[20] Zhang B, Phillips DV, Wu K (1997). Further research on fatigue properties of plain concrete. Mag Concr Res.

[21] Weng CC, Tam MT, Lin GC (1992). Acoustic emission characteristics of mortar under compression. Cem Concr Res.

[22] Yan H, Sun W, Chen H (1999). The effect of silica fume and steel fiber on the dynamic mechanical performance of high-strength concrete. Cem Concr Res.

[23] Sharaky IA, Megahed FA, Seleem MH, Badawy AM (2019). The influence of silica fume, nano silica and mixing method on the strength and durability of concrete. SN Appl Sci.

[24] Nežerka V, Bílý P, Hrbek V, Fládr J (2019). Impact of silica fume, fly ash, and metakaolin on the thickness and strength of the ITZ in concrete. Cem Concr Compos.

[25] Gao Y, Zhou W, Zeng W, Pei G, Duan K (2021). Preparation and flexural fatigue resistance of self-compacting road concrete incorporating nano-silica particles. Constr Build Mater.

[26] Diaz SI, Hilsdorf HK (1971) Fracture mechanics of concrete under static, sustained, and repeated compressive loads. Civil Engineering Studies, SRS No. 382, University of Illinois

[27] Fan Z, Sun Y (2019). Detecting and evaluation of fatigue damage in concrete with industrial computed tomography technology. Constr Build Mater.

[28] Skarżyński Ł, Marzec I, Tejchman J (2019). Fracture evolution in concrete compressive fatigue experiments based on X-ray micro-CT images. Int J Fatigue.

[29] Schaan G, Rybczynski S, Schmidt-Döhl F, Ritter M (2020). Fatigue of concrete examined on the nanoscale—TEM studies of fatigue-induced changes in the cement paste of UHPC. G.I.T. Imaging Microsc.

[30] Shah SG, Kishen JMC (2012). Use of acoustic emissions in flexural fatigue crack growth studies on concrete. Eng Fract Mech.

[31] Finck F (2002). Acoustic emission analysis of SFRC beams under cyclic bending loads. Otto-Graf J.

[32] Scheiden T, Oneschkow N, Löhnert S, Patel R (2019) Acoustic emission due to fatigue damage mechanisms in high-strength concrete with different aggregates. In: Proceedings of SEMC2019: the seventh international conference on structural engineering, mechanics and computation, Cape Town, South Africa, 2–4 September 2019, Alphose Zingoni, Ed.; CRC Press: London, UK. 10.1201/9780429426506

[33] Scheiden T, Oneschkow N (2019). Influence of coarse aggregate type on the damage mechanism in high-strength concrete under compressive fatigue loading. Struct Concr.

[34] Leusmann T, Basutkar G, Lunardelli M, Lowke D (2019) Characterizing the 3D mesostructured of high performance concrete by computed tomography. In: Proceeding of RILEM spring convention and sustainable materials, systems and structures conference, Rovinj, Croatia, 20-22 March 2019, pp 176–184

[35] DIN EN 12390-13:2014-06. Testing hardened concrete—Part 13: determination of modulus of elasticity in compression, German version of EN 12390-13:2013

[36] DIN EN 12390-3:2019-10. Testing hardened concrete—Part 3: Compressive strength of test specimens, German version of EN 12390-3:2019

[37] DIN EN ISO 7500-1:2018-06. Metallic materials—calibration and verification of static uniaxial testing machines—Part 1: tension/compression testing machines—calibration and verification of the force-measuring system, German version of EN ISO 7500-1:2018

[38] Scheiden T, Oneschkow N, Löhnert S, Patel R (2019) Fatigue damage of high-strength concrete with basalt aggregate. In: Proceedings of the fib symposium 2019: concrete—innovations in materials, design and structures, Krakow, Poland, 27–29 May 2019, pp 1896–1903

